# Effect of a Wood-Based Carrier of *Trichoderma atroviride* SC1 on the Microorganisms of the Soil

**DOI:** 10.3390/jof7090751

**Published:** 2021-09-13

**Authors:** Hamza Chammem, Livio Antonielli, Andrea Nesler, Massimo Pindo, Ilaria Pertot

**Affiliations:** 1 Department of Civil, Environmental and Mechanical Engineering, University of Trento, Via Mesiano, 20, 38123 Trento, TN, Italy; hamza.chammem@unitn.it; 2 Center for Health & Bioresources, Bioresources Unit, AIT Austrian Institute of Technology GmbH, Konrad-Lorenz-Strasse 24, 3430 Tulln, Austria; livio.antonielli@ait.ac.at; 3 Bi-PA nv (Biological Products for Agriculture), Technologielaan, 7, B-1840 Londerzeel, Belgium; andrea.nesler@bi-pa.com; 4 Reserch and Innovation Center, Fondazione Edmund Mach (FEM), Via E. Mach 1, 38010 San Michele all’Adige, TN, Italy; massimo.pindo@fmach.it; 5 Center Agriculture Food Environment, University of Trento, Via E. Mach 1, 38010 San Michele all’Adige, TN, Italy

**Keywords:** *Trichoderma*, substrate, soil, metabarcoding, diversity, community composition, wood pellets

## Abstract

Wood pellets can sustain the growth of *Trichoderma* spp. in soil; however, little is known about their side effects on the microbiota. The aims of this study were to evaluate the effect of wood pellets on the growth of *Trichoderma* spp. in bulk soil and on the soil microbial population’s composition and diversity. *Trichoderma atroviride* SC1 coated wood pellets and non-coated pellets were applied at the level of 10 g∙kg^−1^ of soil and at the final concentration of 5 × 10^3^ conidia∙g^−1^ of soil and compared to a conidial suspension applied at the same concentration without the wood carrier. Untreated bulk soil served as a control. The non-coated wood pellets increased the total *Trichoderma* spp. population throughout the experiment (estimated as colony-forming unit g^−1^ of soil), while wood pellets coated with *T.* *atroviride* SC1 did not. The wood carrier increased the richness, and temporarily decreased the diversity, of the bacterial population, with *Massilia* being the most abundant bacterial genus, while it decreased both the richness and diversity of the fungal community. Wood pellets selectively increased fungal species having biocontrol potential, such as *Mortierella*, *Cladorrhinum,* and *Stachybotrys*, which confirms the suitability of such carriers of *Trichoderma* spp. for soil application.

## 1. Introduction

Biological control of soil-borne diseases is a valuable alternative to synthetic chemical fungicides [1], and, within the genus *Trichoderma* [2], several strains have demonstrated good efficacy against soil-borne pathogens such as *Rhizoctonia solani*, *Fusarium* spp., *Pythium* spp., and nematodes of the genus *Meloidogyne* [3]. However, the success of treatments with *Trichoderma* spp. depends highly on the physicochemical and biological traits of the soil, as well as the rhizosphere competence of the strains used. After the soil treatment, the population of *Trichoderma* spp. normally tends to decrease over time [4]. This problem is usually addressed by applying high quantities of the biocontrol agent and/or by formulating the biocontrol agent (i.e., as a wettable powder, emulsion, pellets, granules, etc.) or adding nutrients to the formulation that can extend its longevity in the soil [5,6,7,8]. Particular formulations of biocontrol agents comprise aids that can preserve them, favor their delivery to targets, and improve their activity [9]. Another limiting factor that prevents the widespread use of *Trichoderma* spp. in soil treatments is the difficulty in homogenously applying small quantities of conidia in large volumes of soil [10]. Although several authors have addressed the effectiveness of formulations and the addition of nutrients [11,12,13,14,15,16,17,18,19,20], limited information is available on the effect of such components on the soil microbiome [21]. More particularly, the effect of the use of lignocellulosic substrates inoculated with *Trichoderma* spp. on soil fungal and bacterial populations is unknown.

The use of wood pellets coated with conidia of *Trichoderma* spp. might represent an interesting approach for soil treatments [10]. For example, *Trichoderma atroviride* SC1 can easily grow on beech wood pellets and reach high population levels (e.g., 10^9^ cfu∙g^−1^ of wood pellet), especially if complemented with nitrogen sources, such as soy protein isolates. The advantage of using wood pellets is double: they can be easily spread and incorporated in soil by using standard equipment (e.g., using a fertilizer spreader, followed by harrowing) and support the growth of the fungus, which colonize wood before other microbes and then outcompete them. For example, early or simultaneous inoculation of *T. viride* or *T. harzianum* with basidiomycetes that can attack coconut fibers, such as *Trametes versicolor* and *Stereum rugosum*, can protect coconuts from white rot decay, mainly by nutrient competition, but also by toxins that can inhibit the growth of the pathogens [22]. On the other hand, carriers can also modify the soil microbial communities’ composition. Since soil microbial communities often act synergistically to control soil-borne pathogens, a change in the soil microbial community structure, and/or a reduction in biodiversity, may affect the occurrence of soil-borne diseases [23]. 

Many factors can contribute to shifting the microbial populations in the soil, such as the soil type and pH, structure, salinity, and moisture, but most importantly, soil organic matter and plant exudates [24,25]. Generally, adding organic matter to the soil enhances the microbial activity [26,27,28], while the use of mineral fertilizers can reduce fungal diversity [29,30]. The inoculation of *Trichoderma* spp. without organic matter has a transient effect on the microbial population of the soil [31,32], and combining the application of *Trichoderma* spp. with organic composts and bio-organic fertilizers has been proposed as an alternative to mineral fertilizers; in fact, adding these species to the substrate can increase soil fertility and microbial biodiversity [33,34].

The aim of this study was to test the effectiveness of a carrier of *T. atroviride* SC1 made from wood pellets in prolonging the survival of the fungus in the soil and to evaluate its possible impact on the soil microbiota by metabarcoding analysis of the microbial communities. Although this case study is based on the use of a specific carrier (beech wood pellet) and a specific strain (*T. atroviride* SC1) in a single soil, the protocol could be replicated for other similar combinations, for future comparison.

## 2. Materials and Methods

### 2.1. Coating the Wood Pellets with Trichoderma atroviride SC1, Soil Treatments and Experimental Design

Beech wood pellets (Italwood S.r.l., Piovene Rocchette, Italy) were used as carriers to deliver *T. atroviride* SC1 to the soil. Wood pellets were coated with conidia, according to Chammem et al. [10]. Briefly, beech pellets (100 g) were dried in an oven at 120 °C for 12 h, sprayed with soy protein isolates (30 mg∙mL^−1^ corresponding to a final rate of 3 mg∙g^−1^ of pellets) and coated with 0.1 mL of *T. atroviride* SC1 conidia sterile water suspension (SDW) with a spray bottle, while continuously mixing in a mixer (MUM44R1- BSH Elettrodomestici S.p.A., Milan, Italy) at a speed of 25 rpm. Conidia were prepared according to Longa et al. [35] and adjusted with a hemocytometer at 5 × 10^6^ conidia∙mL^−1^ to reach a final concentration of 5 × 10^5^ conidia∙g^−1^ of pellets. The wood pellets were used immediately after coating.

The experiment was carried out under controlled greenhouse conditions, at a temperature of 25 ± 1°C and relative humidity 70 ± 10%, in 2020. The coated wood pellets were applied to a bulk of sandy loam soil (69.7% sand: 26.3% lime: 4% clay, pH 8) collected in San Michele all’Adige, Italy (N 46.182315, E 11.118804), representing a typical agricultural soil of this region (apple orchards). The soil was mixed thoroughly, sieved, and then distributed into 20 plastic pots (Mongardi, Ferriera di Buttigliera Alta, Italy; 2L) at 1 kg of soil∙pot^−1^. A randomized block design was used, with four treatments and five replicates (pots) each: bulk soil (untreated control; Ctr), soil mixed with non-coated wood pellet (Trt1), soil mixed with a conidial SDW suspension *T. atroviride* SC1 (Trt2), and soil mixed with of *T. atroviride* SC1 coated pellets prepared as described above (Trt3). The final estimated concentration of *T. atroviride* SC1 conidia in Trt2 and Trt3 was 5 × 10^3^ conidia∙g^−1^ of soil. The wood pellets in Trt1 and Trt3 were applied by laying them on the soil surface (10 g∙pot^−1^), spraying 50 mL of SDW pot^−1^, letting them swell and disintegrate (20 min), and gently mixing the broken-down pieces in the soil. The Ctr and Tr2 were sprayed with 50 mL of SDW per pot^−1^. After calculation of the water holding capacity of the soil using the percolation method, the soil was kept at 60 ± 10% humidity, by weighing the pots every two days and adding the quantity of water that was lost by evaporation. The experiment (E1) was repeated after one week (E2).

To ensure the absence of *T. atroviride* SC1 in the soil, real-time PCR primers and probes, designed for the detection and quantification of *T. atroviride* SC1 [36], were used to check the bulk soil before the experiments.

### 2.2. Soil Sampling

The growth of *T. atroviride* SC1 was monitored by sampling the soil immediately after completing the treatments (12 h, D0), every 15 days in the first two months (D15, D30, D45, D60), and the final sampling was carried out after ninety days (D90). Samples of soil (5 g) were collected from each pot (replicate) by taking 1 g from the center of the pot and 1 g from each of its four corners. The samples were put in 50 mL sterile Falcon tubes (Merk Life Science S.r.l., Milan, Italy) and thoroughly mixed. Colony forming unit (cfu) counting was performed by suspending 1 g from each falcon tube in 10 mL of SDW, vortexing for 1 min, and plating 100–200 µL from the SDW suspension on a *Trichoderma* semi-selective medium that contained potato dextrose agar (Oxoid, Basingstoke, UK, 39 g∙L^−1^), rose bengal (Sigma Aldrich, Anekal Taluk, India, 0.1 g∙L^−1^), chloramphenicol (Sigma Aldrich, Beijing, China, 0.1 g∙L^−1^), and streptomycin sulfate (Fluka Biochemika, Milan, Italy, 0.05 g∙L^−1^). The results are reported as cfu∙g^−1^ of soil ± the standard deviation. 

For metabarcoding analysis, only four replicates from each treatment were considered and were chosen randomly. Samples (1 g) were collected at D0, D15, and D90, lyophilized in a freeze-dryer (HetoLyoLab 3000-Analitica De Mori, Milan, Italy) for 12 h, and stored at −80 °C until use.

### 2.3. DNA Extraction, Amplification, and Sequencing

Total genomic DNA was extracted from 500 mg of lyophilized soil samples (96 soil samples) using a FastDNA™ Spin kit (MP Biomedicals, Irvine, CA, USA), following the manufacturer’s instructions and was quantified using a NanoDrop™ 8000 spectrophotometer (Thermo Fisher Scientific, Waltham, MA, USA).

The library construction and sequencing were performed on the sequencing platform of the Edmund Mach Foundation. Total genomic DNA was amplified using primers specific to either the bacterial and archaeal 16S rRNA gene or the fungal ITS1 region. The specific bacterial primer set 515F (5′-GTGYCAGCMGCCGCGGTAA-3′) and the 806R (5′-GGACTACNVGGGTWTCTAAT-3′) was used [37], with degenerate bases suggested by Apprill et al. [38] and by Parada et al. [39]. Although no approach based on PCR amplification is free from bias, this primer pair has been shown to guarantee good coverage of known bacterial and archaeal taxa [40]. For the identification of fungi, the internal transcribed spacer 1 (ITS1) was amplified using the primers ITS1F (5′-CTTGGTCATTTAGAGGAAGTAA-3′) [41] and ITS2 (5′-GCTGCGTTCTTCATCGATGC-3′) [42]. All the primers included specific overhang Illumina adapters for the amplicon library construction.

For the 16S V4 region, each sample was amplified by PCR using a 25-μL reaction with one μM of each primer. In more detail, 12.5 μL of 2× KAPA HiFi HotStart ReadyMix and 10 μL forward and reverse primers were used in combination with 2.5 μL of template DNA (5–20 ng∙μL^−1^). PCR reactions were executed using a GeneAmp PCR System 9700 (Thermo Fisher Scientific) and the following cycling conditions: initial denaturation step at 95 °C for 5 min (1 cycle); 28 cycles at 95 °C for 30 s, 55 °C for 30 s, and 72 °C for 30 s; a final extension step at 72 °C for 5 min (1 cycle).

For the ITS1 region, each sample was amplified by PCR using 25-μL reaction with 10 μM of each primer. In more detail, 22 μL of premix FastStart High Fidelity PCR System (Roche) and 2 μL forward and reverse primers were used in combination with 1 μL of template DNA (5–20 ng∙ul^−1^). PCR reactions were executed using a GeneAmp PCR System 9700 (Thermo Fisher Scientific) and the following cycling conditions: initial denaturation step at 95 °C for 3 min (1 cycle); 30 cycles at 95 °C for 20 s, 50 °C for 45 s, and 72 °C for 90 s; final extension step at 72 °C for 10 min (1 cycle).

The amplification products were checked on 1.5% agarose gel and purified using a CleanNGS kit (CleanNA, Waddinxveen, The Netherlands), following the manufacturer’s instructions. Afterward, a second PCR was used to apply dual indices and Illumina sequencing adapters Nextera XT Index Primer (Illumina, Berlin, Germany), using seven cycles of PCR (16S Metagenomic Sequencing Library Preparation, Illumina, Berlin, Germany). The amplicon libraries were purified using a CleanNGS kit (CleanNA, Waddinxveen, The Netherlands), and quality control was performed on a Typestation 2200 platform (Agilent Technologies, Santa Clara, CA, USA). Finally, all barcoded libraries were pooled in an equimolar manner and sequenced on an Illumina^®^ MiSeq (PE300) platform (MiSeq Control Software 2.5.0.5 and Real-Time Analysis software 1.18.54.0).

### 2.4. Bioinformatics and Statistical Analysis

Illumina reads were filtered with Bowtie2 v2.3.4.3 [43] to avoid the presence of Illumina phiX contamination, and quality was preliminarily checked with FastQC v0.11.8 [44]. Primers were stripped using Cutadapt v1.18 [45]. Sequences were quality filtered, trimmed, denoised, and amplicon sequence variants (ASVs) were generated with DADA2 v1.14 [46]. Denoised forward and reverse ASV sequences were merged and chimeras were removed. Filtered ASVs were checked using Metaxa2 v2.2.1 [47] and ITSx v1.1.2 [48] for targeting the presence of the V4 16S rRNA and ITS1 regions, in archaeal and bacterial sequences and fungal sequences, respectively. Taxonomic assignment of 16S rRNA gene ASVs and ITS based ASVs was performed using a RDP classifier, reimplemented in DADA2 against the SILVA v138 database [49] and UNITE 8.2 database [50], respectively. BIOM objects with bacterial and fungal counts, respectively, were built and imported into the R-4.0.3 statistical environment for further analyses [51].

**Figure 1:**
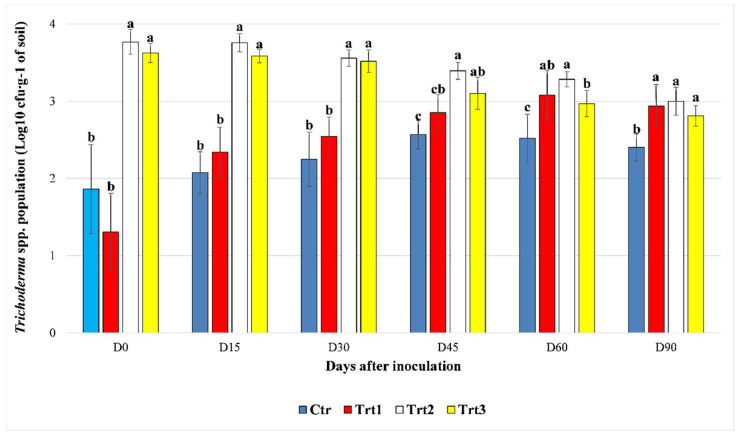
The effect of beech wood pellets coated with *Trichoderma atroviride* SC1 and uncoated on the *Trichoderma* spp. population in the soil at different sampling times post inoculation (D0: after 12h, D15: after 15 days, D30: after 30 days, D45: after 45 days, D60: after 60 days, and D90: after 90 days). Ctr: bulk soil; Trt1: soil with 10 g of beech wood pellets; Trt2: soil with *T. atroviride* SC1 conidial suspension (5 × 10^3^ conidia∙g^−1^ of soil); Trt3: soil with 10 g of *T. atroviride* SC1 coated beech pellets (5 × 10^5^ conidia∙g^−1^ of beech wood pellets). At each sampling point, different letters indicate significant statistical differences between treatments, according to Dunn’s test (α = 0.05). Colony counts of the two experiment were pooled.

The data of the growth assessment of *T. atroviride* SC1 cfu counts (Figure 1) were log10 transformed to simplify the data analysis, as is commonly the case for colony counts to avoid data skewness. Bartlett’s test of homogeneity of variances and Shapiro–Wilk’s normality test were used to check the normal distribution of the data. ANOVA and Tukey’s HSD tests were performed on log10 transformed data with a normal distribution (test of the evolution of treatments in time), and the non-parametric Kruskal–Wallis and Dunn post-hoc (Benjamini–Hochberg *p*-adjustment method α = 0.05) tests were used otherwise (comparing the cfu counts between treatments at each sampling point).

Bacterial and fungal count tables were filtered using the RAM R package, and rare ASVs (relative abundance < 0.1%) were discarded. Relative abundance of taxa at different taxonomic ranks was calculated with the RAM R package [52]. 

**Figure 2:**
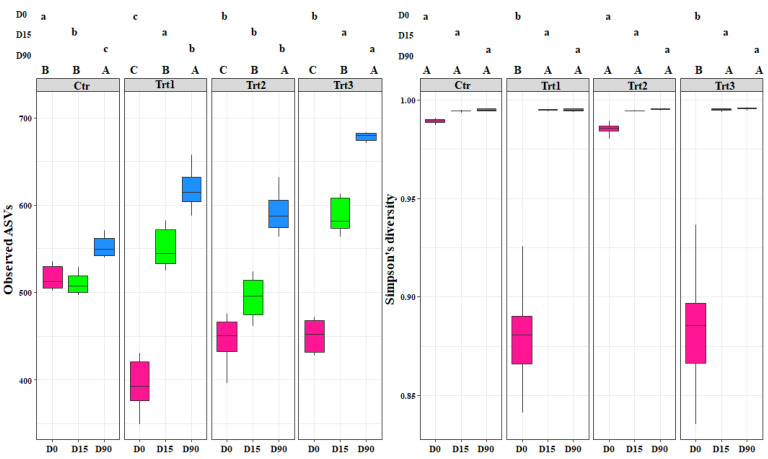
Boxplots of the bacterial richness (observed ASVs) and bacterial diversity (Simpson’s diversity) at different sampling times (D0: after 12 h; D15: after 15 days; D90: after 90 days). Ctr: bulk soil; Trt1: soil with 10 g of beech wood pellets; Trt2: soil with *Trichoderma atroviride* SC1 conidial suspension at the rate of 5 × 10^3^ conidia∙g^−1^ of soil; Trt3: soil with 10 g of *T. atroviride* SC1 coated beech pellets (5 × 10^5^ conidia∙g^−1^ of beech wood pellets). Letters indicate significant differences according to the emmeans package (α = 0.05) between treatments at each sampling point (lower case letters) or for the same treatment over time (upper case letters). Data of the two experiments E1 and E2 were pooled.

**Figure 3:**
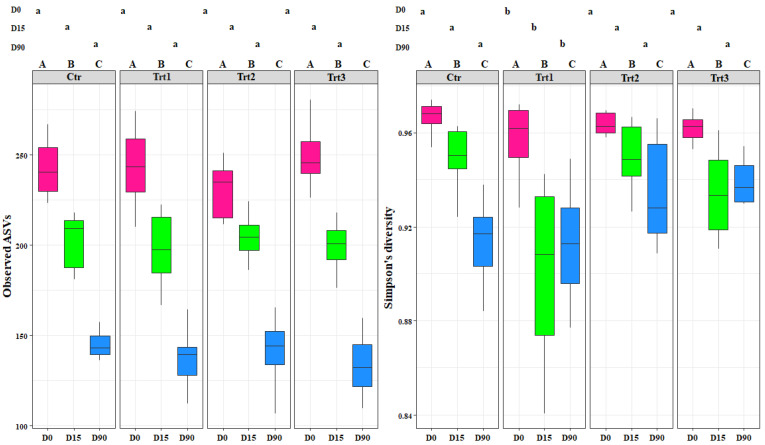
Boxplots of the fungal richness (observed ASVs) and fungal diversity (Simpson’s diversity) at different sampling times (D0: after 12 h; D15: after 15 days; D90: after 90 days). Ctr: bulk soil; Trt1: soil with 10 g of beech wood pellets; Trt2: soil with *Trichoderma atroviride* SC1 conidial suspension at the rate of 5 × 10^3^ conidia∙g^−1^ of soil; Trt3: soil with 10 g of T. *atroviride* SC1 coated beech pellets (5 × 10^5^ conidia∙g^−1^ of beech wood pellets). Letters indicate significant differences according to the emmeans package (α = 0.05) between treatments at each sampling point (lower case letters) or for the same treatment over time (upper case letters). Data of the two experiments E1 and E2 were pooled.

Alpha-diversity values were calculated adopting a multiple rarefaction method, implemented in the rtk R package [53]. In detail, richness (observed ASVs) and diversity values (Simpson’s index) were generated by averaging the results inferred after 999 rarefactions, starting from a minimum number of 38,256 and 13,418 reads, for 16S rRNA gene and ITS data, respectively. A regression analysis based on linear models was carried out on the richness and diversity values, for each dataset, after inspection with the fitdistrplus R package [54]. In more detail, a machine learning approach based on 9999 bootstrap resampling was adopted to evaluate models in which factors (i.e., experiment, time, and treatment) were considered only for their main effects or also with an interaction. The performance of the models was assessed by means of RMSE (root mean squared error) and R-squared, which measure the prediction error and the proportion of variation explained by each model, respectively [55]. An analysis of variance (ANOVA) followed, to evaluate the linear model fit. A post-hoc analysis was carried out with pairwise comparisons, based on the estimated marginal means (EMMs) as implemented in the emmeans R package [56]. Richness and diversity values were graphically represented as boxplots, using the ggplot2 R package (Figure 2 and Figure 3) [57]. A confirmatory analysis based on recursive partitioning [58] was carried out by considering richness and diversity variables together in the same model; with experiment, time, and treatment as factors (Figure S1).

**Figure 4:**
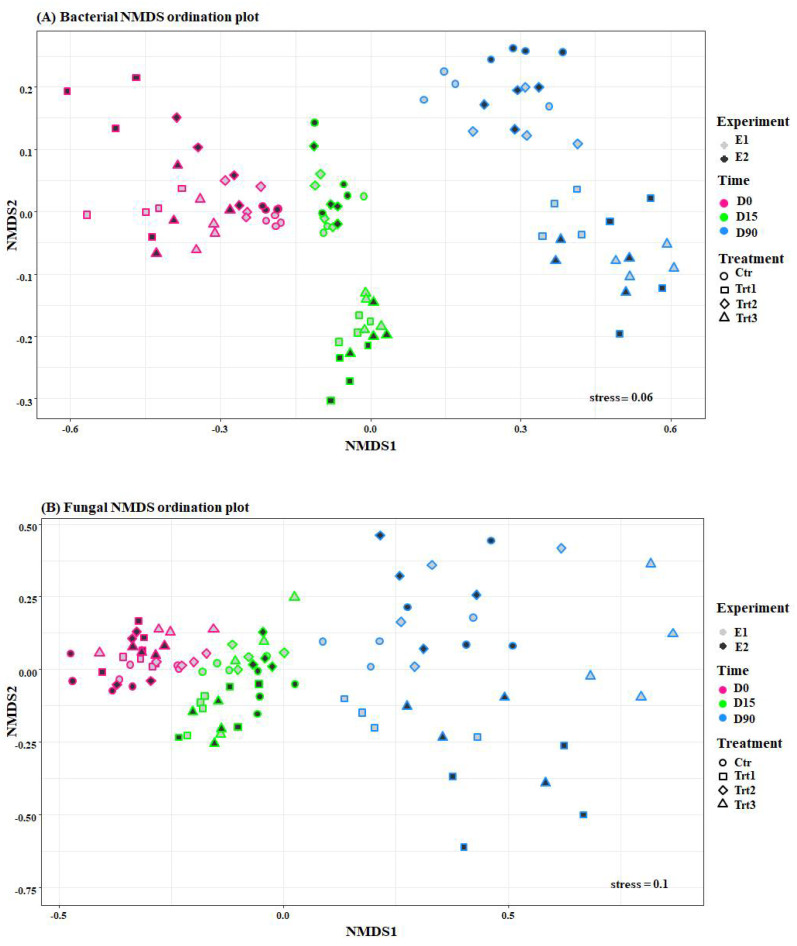
Ordination plots of non-metric multidimensional scale analysis (NMDS) using the Bray-Curtis dissimilarities of bacterial (**A**) and fungal (**B**) communities. Pink, green, and blue colors indicate different sampling times (D0: after 12 h; D15: after 15 days; D90: after 90 days) and show how both fungal and bacterial communities are grouped by time. The colors of the filling, grey and black, represent the two experiments (E1 and E2, respectively), and different shapes represent the treatments that grouped, mainly the bacterial community. Ctr: bulk soil; Trt1: soil with 10 g of beech wood pellets; Trt2: *Trichoderma atroviride* SC1 applied to the soil as a conidial suspension at the rate of 5 × 10^3^ conidia g^−1^ of soil; Trt3: soil with 10 g of *T. atroviride* SC1 coated beech wood pellets at 5 × 10^5^ conidia g^−1^.

**Figure 5:**
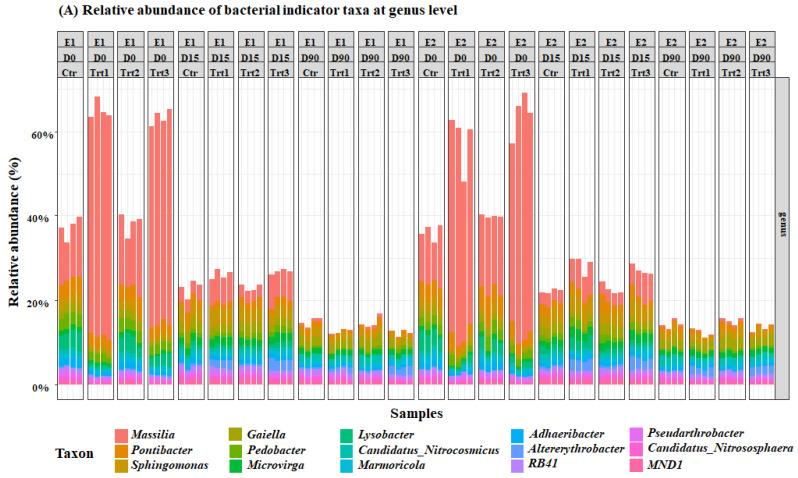

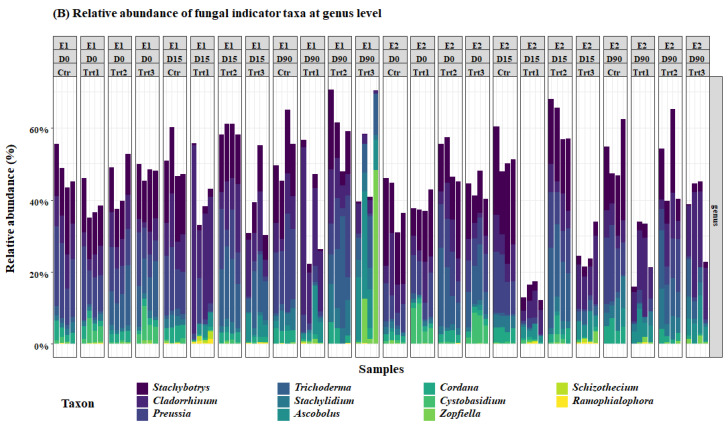
Relative abundance of the most important bacterial (**A**) and fungal (**B**) genera. E1and E2: two experiments with four treatments at different sampling times (D0: after 12 h; D15: after 15 days; D90: after 90 days); Ctr: bulk soil; Trt1: soil with 10 g of beech wood pellets; Trt2: soil with *Trichoderma atroviride* SC1 conidial suspension at the rate of 5 × 10^3^ conidia∙g^−1^ of soil; Trt3: soil with 10 g of T. *atroviride* SC1 coated beech pellets (5 × 10^5^ conidia∙g^−1^ of beech wood pellets). Data of each replicate are presented in the histograms.

Beta diversity calculations were conducted after normalization with the median of ratios method implemented in the DESeq2 R Bioconductor package [59]. Exploratory non-metric multidimensional scaling (NMDS) ordinations were applied to Bray–Curtis dissimilarities. NMDS ordinations were plotted using the ggvegan and ggplot2 R packages (Figure 4) [60]. A multivariate analysis based on PERMANOVA was performed on Bray–Curtis dissimilarities applied to normalized bacterial and fungal count tables, respectively (adonis function, vegan R package) [61]. To confirm the PERMANOVA results, a multivariate generalized model (mGLM) was calculated, including all available factors and based on a negative binomial distribution (confirmed by graphical inspection). The model was assessed by analysis of deviance with a likelihood-ratio-test (manyglm function, mvabund R package) [62]. ASVs that had abundances significantly different (*p* < 0.05) in at least one factor were extracted from the mGLM results and were used to calculate univariate non-parametric tests for each factor (multtest.gp function, RVAideMemoire R package) [63]. The results of each rank test were corrected by false discovery rate (FDR), and post-hoc pairwise comparisons were performed between the levels in each factor, with a Dunn test followed by Benjamini–Hochberg adjustment (dunntest function, FSA R package) [64]. Bacterial and fungal indicator ASVs, respectively, were collapsed at genus level and relative abundances were plotted with the RAM R package (Figure 5).

## 3. Results

### 3.1. Impact of the Trichoderma atroviride SC1 Coated Beech Wood Carrier on the Growth of Trichoderma spp. in Soil

*Trichoderma atroviride* SC1 DNA was not detected in the original bulk soil. Since the cfu counting does not allow for distinguishing the species/strains of *Trichoderma*, *T. atroviride* SC1 and the indigenous population of *Trichoderma*, are mentioned as *Trichoderma* spp. population throughout the paper.

The *Trichoderma* spp. cfu counts increased rapidly in the first 30 days, until D45 in Ctr and Trt1. For Ctr, the cfu counts were not significantly different at D15 and D30 compared to D0, according to Dunn’s post-hoc test (*p* = 0.380; *p* = 0.080). Then at D45, the counts reached levels significantly different from the ones registered at D0 and D15 (*p* = 0.001; *p* = 0.002). No significant difference was observed between the cfu counts of the *Trichoderma* spp. population at D60 compared to D30 (*p* = 0.074) and D45 (*p* = 0.370), respectively, while it remained significant for D0 (*p* = 0.002) and D15 (*p* = 0.004). At the end of the experiment (D90), a slight decrease in the population was observed (2.69 × 10^2^ ± 102.87 cfu∙g^−1^ of soil) and a significant difference was recorded only with D0 (*p* = 0.021). For Trt1, the colonies started to grow faster than Ctr, and a significant difference was detected starting from D30 as compared to D0 (*p* = 0.010). The population of *Trichoderma* spp. continued to grow and became significantly different from the levels observed at D30 a month later at D60 (*p* = 0.009). The levels registered at D90 (1.00 × 10^3^ ± 712.57 cfu∙g^−1^ of soil) remained significantly different from the cfu counts at D30, but not different from those of D45 (*p* = 0.275) and D60 (*p* = 0.338). The treatments Trt2 and Trt3, where *T. atroviride SC1* was inoculated at the rate of 5 × 10^3^ conidia∙g^−1^ of soil, maintained the same level of cfu count in the first 30 days compared to D0, with *p* = 0.065 and *p* = 0.206 for Trt2 and Trt3, respectively. Then at D45, the population of *Trichoderma* spp. started to decline in Trt2 (2.54 × 10^3^ ± 689.00 cfu∙g^−1^ of soil) and Trt3 (1.40 × 10^3^ ± 681.09 cfu∙g^−1^ of soil), reaching levels significantly different from the ones registered at D15 (*p* = 0.003; 0.001), and the same was observed between D60 and D30 (*p =* 0.012; *p =* 0.001). The population at D90 was significantly lower than all the other sampling points for Trt2, except for D60 (*p* = 0.103), while no significant difference was observed between D45, D60, and D90 for Trt3 (*p* = 0.057; *p* = 0.218). 

Between treatments, at D0, there was a significant difference in the cfu counts between Ctr/Trt1 and Trt2/Trt3 (H = 31.538, df = 3, *p* < 0.001), which persisted until D45. At D45, no significant difference was observed between the counts of Ctr and Trt1 (*p* = 0.052), Trt1 and Trt3 (*p* = 0.054), or Trt2 and Trt3 (*p* = 0.050). Trt2 and Trt3 were both significantly different from Ctr (H = 29.067, df = 3, *p* < 0.001). Trt2 registered the highest cfu count among the treatments (2.54 × 10^3^ ± 689.82 cfu∙g^−1^ of soil).

At D60 the cfu counts of Trt1 continued to rise and became significantly different from the control (*p* = 0.003). The cfu counts in Trt3 continued to decrease and became significantly different from the cfu counts in Trt2 (*p* = 0.011).

At D90, the population of *Trichoderma* spp. showed no statistical difference (*p* = 0.250; *p* = 0.140; *p* = 0.040) between Trt1 (1.03 × 10^3^ ± 712.71 cfu∙g^−1^ of soil), Trt2 (1.07 × 10^3^ ± 417.05 cfu∙g^−1^ of soil), and Trt3 (6.76 × 10^2^ ± 232.81 cfu∙g^−1^ of soil), with cfu counts that were significantly higher than the control (H = 23.766, df = 3, *p* < 0.001) (Figure 1).

### 3.2. Impact of the Trichoderma atroviride SC1 Coated Beech Wood Carrier on the Microorganisms of the Soil

A total of 103,970.6 bacterial/archaeal reads and 86,077.48 fungal reads were obtained. The most dominant bacterial phyla, in terms of relative abundance, were Proteobacteria (34%), Crenarchaeota (10%), Actinobacteriota (10%), Bacteroidota (10%), and Acidobacteriota (10%). At genus level, *Massilia* was the most abundant, with 12%, followed by *Pontibacter*, *Sphingomonas*, and *Gaiella,* with (2%) and finally *Microvirga* (1%). The fungal taxa were dominated by Ascomycota (82%), then Basidiomycota (6%), and Mortierellomycota (6%), followed by Chytridiomycota (3%) and Aphelidiomycota (3%). *Mortierella* (6%) was the most dominant fungal genus, followed by *Fusarium* (4%) and *Cladorrhinum* (4%), and finally *Gibberella* (3%), then *Stachybotrys* (2%).

#### Bacterial and Fungal Richness and Diversity

The bacterial alpha diversity showed statistical differences in richness (observed ASVs) between the different sampling time points for each treatment (*F* = 514.48, *p* < 0.001) and between treatments (*F* = 45.94, *p* < 0.001). The increase in the bacterial richness occurred faster for all treatments (15 days after the inoculation) compared to the control, which showed a significant increase after 90 days (Figure 2). This shows that the introduction of the carrier components, the incorporation of *T. atroviride* SC1 into the soil, and their combined application enhanced the bacterial richness. At D90, the highest effect was observed with Trt3, which presented the highest ASVs (680) among all treatments, while no significant difference was observed between Trt1 and Trt2.

In contrast, the bacterial diversity (Simpson’s index) showed differences between treatments at D0 only (*F* = 245.07, *p* < 0.001). The treatments where a carrier was applied with or without *T. atroviride* SC1 (Trt1 and Trt3) affected the bacterial population community and decreased its diversity, as is shown by the low Simpson’s values (0.88 and 0.87) for Trt1 and Trt3, respectively. The addition of the carrier, which contains carbon and nitrogen sources, clearly favored certain genera that are more adapted to those components and decreased the less competent ones (Figure 2). Generally, the introduction of *T. atroviride* SC1 did not affect the bacterial population and the most important factor that governed the bacterial dynamics was the addition of the carrier (*F* = 61.40, *p* < 0.001).

The fungal alpha diversity showed significant differences in richness between the different sampling time points for each treatment (*F* = 303.58, *p* < 0.001), but not between treatments (*F* = 0.31, *p* = 0.81). The richness decreased in time for both control and treatments, with no exceptions (Figure 3). 

Simpson’s diversity index was significantly different for Trt1 compared to the other treatments, including the control (*F* = 5.17, *p* < 0.001) (Figure 3). This shows the effect of combining the addition of wood to the soil with the application of *T. atroviride* SC1. The fungal diversity in the treatments where *T. atroviride* SC1 was applied with and without wood pellets were not significantly different from the control (Figure 3). In contrast, the fungal diversity in Trt1, which was supplemented only with wood, was significantly different. This shows that adding *T. atroviride* SC1 can counterbalance the effects of the wood on the fungal diversity. The addition of wood was the main cause for the differences observed between treatments. The recursive partitioning analysis of the bacterial and fungal richness and diversity confirmed the above-mentioned results (Figure S1).

Unsupervised non-metric multidimensional scaling (NMDS) ordinations applied on Bray–Curtis dissimilarities showed that the dissimilarities observed among the bacterial samples were grouped (Figure 4) according to the factors, time and treatment, while it pooled the fungal communities only according to the factor, time (Figure 4). In fact, the permutational multivariate analyses of variance (PERMANOVA) on Bray–Curtis dissimilarities revealed that time was responsible for the biggest portion of the variation in microbiome beta-diversity. The bacterial community differed very significantly according to the factor time (*F* = 144.95, R^2^ = 0.56, *p* < 0.001) and treatment (*F* = 20.38, R^2^ = 0.11, *p* < 0.001), as well as their interaction (*F* = 11.37, R^2^ = 0.13, *p* < 0.001), and less significantly with the factor experiment (*F* = 3.47, R^2^ = 0.006, *p* = 0.015). The same was observed for the fungal communities, which varied according to time (*F* = 21.99, R^2^ = 0.23, *p* < 0.001), treatment (*F* = 8.59, R^2^ = 0.14, *p* < 0.001), the interaction between the two factors (*F* = 3.35, R^2^ = 0.10, *p* = 0.001), and finally the factor experiment (*F* = 2.64, R^2^ = 0.014, *p* = 0.006). These results indicate a high consistency of the effects of the treatments over time (*T. atroviride* SC1 coated and uncoated wood pellets and *T. atroviride* SC1) on soil microbial communities.

Bacterial and fungal indicator ASVs that have significantly different abundances (*p* < 0.05) in the factor time and treatment were extracted from the mGLM results. The results of the Simpson’s bacterial diversity index correspond with an increase in the population of the genus *Massilia,* which was significantly different between the two groups Ctr/Trt2 and Trt1/Trt3 (Figure 5). The results also revealed a significant increase in terms of the relative abundances of the genera *Pontibacter*, *Sphingomonas*, *Gaiella*, *Pedobacter*, and *Microvirga* (Figure 5).

For the fungal community, at D0, *Trichoderma* spp. were higher in Trt2 and Trt3 than in Ctr and Trt1, as expected. Contrarily to the cfu counts, however, this did not change until the end of the experiment, according to Dunn’s post-hoc test. The carrier (Trt1/Trt3) selectively and significantly increased the total ASV’s of *Cystobasidium*, *Ascobolus*, *Stachybotrys*, *Cladorrhinum*, *Preussia*, and *Stachylidium* (Figure 5).

## 4. Discussion

*Trichoderma* spp. formulations are important, as they can delay the decline of the population of the fungus, protecting conidia from soil fungistasis [65,66], by providing nutrients to selectively stimulate their growth, or by combining both mentioned benefits [67]. In our experiment, we tested the effect of a carrier based on lignocellulosic materials (beech wood pellet supplemented with soy protein isolates) on the growth of *T. atroviride* SC1 in a sandy loam, and assessed the effect of such a carrier on the microbiota of the soil. Since it was hard to distinguish colonies of our strain from the indigenous population in the soil, we reported the results as the population of the total *Trichoderma* spp., instead of *T. atroviride* SC1.

The *Trichoderma* population showed a steady growth in the Ctr and Trt1 for the first 30 to 45 days, then it stabilized. This growth can be explained by the addition of SDW, which stimulated the indigenous population to germinate and exploit the soil organic matter. This is in concordance with studies that show that water affects the microbial growth in the soil [68,69,70]. Trt2, where *T. atroviride* SC1 was applied as an SDW conidial suspension, showed the typical population decline that is reported in literature when *Trichoderma* spp. are applied as conidia [4,35]. The population remained stable for 30 days, then started to decline gradually until D90. This result is in concordance with [31], who reported a decrease in the population of *Trichoderma atroviride* I-1237 after three weeks in neutral clayey soils and after 13 weeks after its inoculation into an acidic sandy loam. Their research showed that the decline of the population of *Trichoderma* spp. can be governed by the physicochemical characteristics of a soil. In fact, since the growth of *T. atroviride* SC1 on wood has been demonstrated by previous studies [10,71], we expected the fungus to grow to higher levels in Trt3 compared to Trt1 and Trt2; however, surprisingly, the total *Trichoderma* population remained stable for 30 days and then declined to levels similar to the ones registered with Trt1 and Trt2, which suggests a soil fungistatic effect that inhibited the growth of *T. atroviride* SC1. The growth of *T. atroviride* SC1 in the soil can be hindered by unfavorable soil conditions (pH 8 in our experiment), as it grows best in acidic conditions [72]. This has also been reported in other studies, which showed that the growth of *Trichoderma* spp. can be affected by soil texture and pH [73,74,75].

The carrier increased the *Trichoderma* spp. population in Trt1 steadily in the first 45 days and then it stabilized to levels that were not significantly different to those observed in the treatments where *T. atroviride* SC1 was added with and without coated beech pellets. Since Trt1 did not contain detectable levels of *T. atroviride* SC1 (since initially we tested the soil for the presence of this fungal strain using specific primers [36]) the presence of other competitive strains of *Trichoderma* spp. that are more efficient than our strain in degrading wood is highly plausible. Competition with other *Trichoderma* spp. could have played an important role in slowing down the growth of *T. atroviride* SC1 with the carrier in Trt3 as compared to Trt1, where the population of *Trichoderma* flourished in the first 45 days. Generally, *Trichoderma* spp. compete with each other in the soil for wood colonization with an effectiveness that depends on the species [76,77]. Several species have been reported in the literature for their high cellulolytic activity, such as *T. reesei*, *T. viride*, *T. harzianum*, *T. virens,* and *T. longibrachiatum* [78,79,80]. The difference that was observed between the results of the cfu counts of *Trichoderma* spp. and the results of the ITS amplicon-based analysis is in concordance with previous studies, which reported that such differences could be due to the fact that a dead propagule that still contains DNA does not develop into a cfu [81]. 

Since soil experiments can be influenced by a complex of physicochemical factors of a soil, such as temperature, texture, water availability, aeration, and light, as well as other biological factors, consisting mainly of the distribution of microorganisms in the soil and their interactions [69], we repeated the experiment twice. Our results show that the experimental design consistently detected the differences occurring due to time and treatment. The incorporation of *T. atroviride* SC1 did not affect the bacterial richness and diversity. This is in concordance with other studies that reported a transient effect of *Trichoderma* spp. on the microbial population [31,36,82,83]. The fungal richness, however, decreased in all treatments, probably due to the growth of fungal genera that are more competitive in growing in conditions either of low organic matter or on woody substrates. Cordier and Alabouvette [31] observed the same effect with the introduction of *T. atroviride* I-1237 on the fungal community; however, the change only lasted for three months, which was the full length of our experiment. The fungal diversity also decreased in all treatments; however, it decreased the most in Trt1, where pellets were introduced in the absence of *T. atroviride* SC1. This suggests that the main driver of the change observed between treatments was the introduction of non-coated beech wood pellets and that *T. atroviride* SC1 contributed in balancing the fungal diversity, probably by increasing the availability of nutrients to other fungi. This is in concordance with Asghar and Kataoka [34], who found that introducing organic amendments into the soil had a negative effect on the diversity of the fungal community. Longa et al. [84] also reported a correlation between the increase in the organic matter and the abundance of microbial functional groups with agricultural importance, such as nitrogen fixing bacteria when soil was supplied with green manure.

In our study, the tested soil was rich in wood-degrading bacteria and fungi in all treatments, as was demonstrated by the analysis of the top ten genera of bacteria and fungi, in terms of relevant abundance. These analyses revealed a dominance of the genus *Massila* for bacteria in all treatments, including the control. *Massilia,* which are saprophytic and opportunistic and were significantly more abundant in the presence of the carrier at D0. This can be explained by the early growth of these bacteria on wood pellets. In fact, *Oxalobacteraceae* in general, and *Massilia* spp. in particular, are very active in the early stages of bacterial succession in soils [85], and species of this genus can produce cellulases [86,87], which can explain their initial growth on wood and soil amended with fresh plant residues [88,89]. However, this effect is usually transient and only occurs when sufficient carbon and energy sources are present, and before competition with other microorganisms becomes limiting [87]. Overall, the carrier increased the bacterial richness and had a transient effect on its diversity. This is in concordance with other studies that showed that using inorganic nitrogen fertilizers in bulk soils affects their bacterial composition [90]. Illescas et al. [91] found that inorganic fertilizers increased bacterial genera with antagonistic activities, such as *Sphingomonas* and *Pseudomonas*, *Kaistobacter*, and *Streptomyces*. We observed the same pattern with *Sphingomonas* and *Pseudomonas*, but also *Lysobacter,* which was not reported in their study. *Lysobacter* spp., named after their lytic effects on other microorganisms and which are often good biocontrol agents, are Gram-negative bacteria that are frequently found in soils. Their increase could be a response to the increase of other microorganisms or to the availability of a nutritional substrate related to the treatments [92].

The carrier also enriched the presence of bacteria involved in the soil nitrogen cycle such as *Microvirga* and *Pedobacter,* which confirms the results obtained by Longa et al. [84] when soil was supplemented with green manure. These results suggest a selective effect of a wood-based carrier of *T. atroviride* SC1. This selective effect was more visible for fungi. In fact, non-coated beech wood pellets decreased both the richness and diversity of the fungal population. This could be the result of promoting fungi that are more adapted to the addition of wood, particularly in bulk soils [90], such as *Mortierella,* which was the most abundant genus. These fungi are considered good degraders of toxic pollutants, such as pesticides and heavy metals [91,93,94], and they have potential for biocontrol [95,96,97]. They are also important plant growth promoters and can enhance soil conditions under salt stress [98,99], but most importantly, they can degrade cellulose, hemicellulose, and organic matter in periods that range from 30 to 430 days, depending on the substrate and the soil conditions [100,101]. Tamayo-Vélez et al. [100] reported an optimal degradation rate of organic matter by *Mortierella* 90 days after inoculation. These results can be compared to what we have observed, as 90 days were sufficient to rank *Mortierella* spp. as the most abundant fungi in the soil. This shows that wood pellets are a suitable substrate for the growth of *Mortierella* spp. This might have caused the competition that prevented *Trichoderma* spp. in general, and *T. atroviride* SC1 in particular, from thriving in the pots supplemented with coated wood pellets. 

Another genus that might have played an important role in the competition for wood degradation is *Cladorrhinum*. *Cladorrhinum* spp. have been extensively reported in agricultural soils [102,103]. They were found in soil as saprotrophs on dung or plant material [104,105], or in roots as endophytes [106]. It is an ammonia fungus belonging to the early successional phase of fungi involved in the saprotrophic litter decomposition in soil [107]. Moreover, they are more efficient than *Trichoderma* for the degradation of hardwood [23,108]. This selective abundance of the carrier might be beneficial, as Gasoni and Stegman de Gurfinkel [109] reported a potential antagonistic activity of *Cladorrhinum* spp. against *Rhizoctonia solani*. In fact, this is not the only fungus found in our research that can be effective against *Rhizoctonia solani*. Some species of the genus *Stachybotrys*, which is also commonly found in soils and on cellulose [110,111], have a strong antagonistic activity against *R. solani* through mycoparasitism, by the means of chitinases and β-1,3-glucanases [110]. One of the species of this genus, namely, *Stachybotrys chartarum* was also suspected to play a role in the development of human pulmonary diseases; however, the results are not yet conclusive [112,113]. Generally, drying pellets before coating, as suggested in our protocol, would eliminate any potential development of unwanted molds that could be harmful to animals or humans; however, further research is needed in different soil types and with different wood species. The results of the selective abundance of beneficial microbes is in concordance with Sani et al. [114], Fu et al. [115], and Zhang et al. [99], who reported a decrease in the relative abundance of genera hosting phytopathogens such as *Neonectria* and *Fusarium,* improved soil fertility, and an increase in the relative abundance of plant growth-promoting rhizobacteria, respectively, when different *Trichoderma* isolates where inoculated into the soil. In our research, the results of the two experiments yielded more consistent results with bacteria compared to fungi. This could be explained by the high competition between fungi in colonizing the woody substrate. The decreased prevalence of wood degrading fungi in some samples was replaced by an increase in the relative abundance of the genera *Cordana*, *Cystobasidium*, *Zopfiella*, *Schizothecium,* and *Ramophialophora*, which might be due to the heterogeneous distribution of microorganisms in the soil [69].

## 5. Conclusions

The incorporation of *T. atroviride* SC1 coated wood pellets in the soil enriched the bacterial community and had a selective effect on the abundance of beneficial fungal species that have biocontrol potential. Although a wider screening of a combination of different wood and soil types is necessary to confirm this effect, these results are promising as they reinforce the suitability of the use of wood pellets as carriers of *Trichoderma* spp., because, in addition to being good growth substrates for the fungus, they have a mild effect on the microbial community, as they induce temporary side effects on the bacterial community, and favor the growth of fungal species with biocontrol potential. In addition, the carrier did not increase any potential human or plant pathogens. Although wood pellets can be used as carriers of conidia of *Trichoderma* spp., further studies are needed to assess their efficacy against soil-borne pathogens and to optimize application conditions and dosages in order to reach an efficient biocontrol effect. Possible side-effects on the plant, e.g., phytotoxicity, must also be tested.

## Data Availability

Not applicable.

## Supplementary Materials

The following are available online at https://www.mdpi.com/article/10.3390/jof7090751/s1, Figure S1: Recursive partitioning analysis of bacterial richness and diversity.
