# Supplementary material for: Effect of a Wood-Based Carrier of Trichoderma atroviride SC1 on the Microorganisms of the Soil
Source: J Fungi (Basel). 2021 Sep 13;7(9):751. doi: 10.3390/jof7090751 (PMC8467423; doi:10.3390/jof7090751)
Supplement: Supplementary file 1 [file jof-07-00751-s001.zip › jof-1363365-supplementary.pdf]

Fig. S1

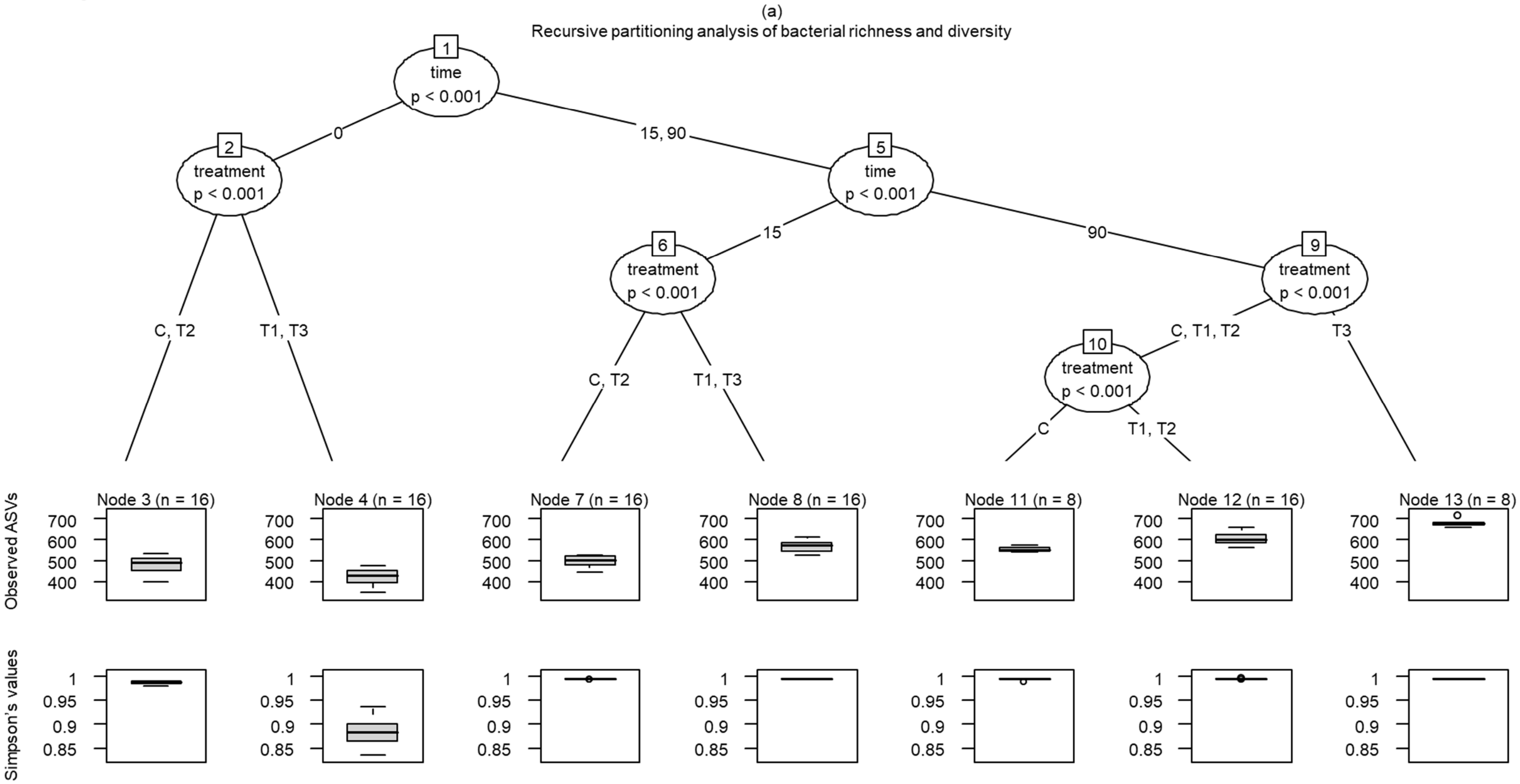
(a)
Recursive partitioning analysis of bacterial richness and diversity
1
time
p < 0.001
0
15, 90
2
treatment
p < 0.001
5
time
p < 0.001
15
90
6
treatment
p < 0.001
9
treatment
p < 0.001
C, T2
T1, T3
C, T2
T1, T3
C, T1, T2
T3
10
treatment
p < 0.001
C
T1, T2
Node 3 (n = 16)
Node 4 (n = 16)
Node 7 (n = 16)
Node 8 (n = 16)
Node 11 (n = 8)
Node 12 (n = 16)
Node 13 (n = 8)
Observed ASVs
700
600
500
400
Simpson's values
1
0.95
0.9
0.85

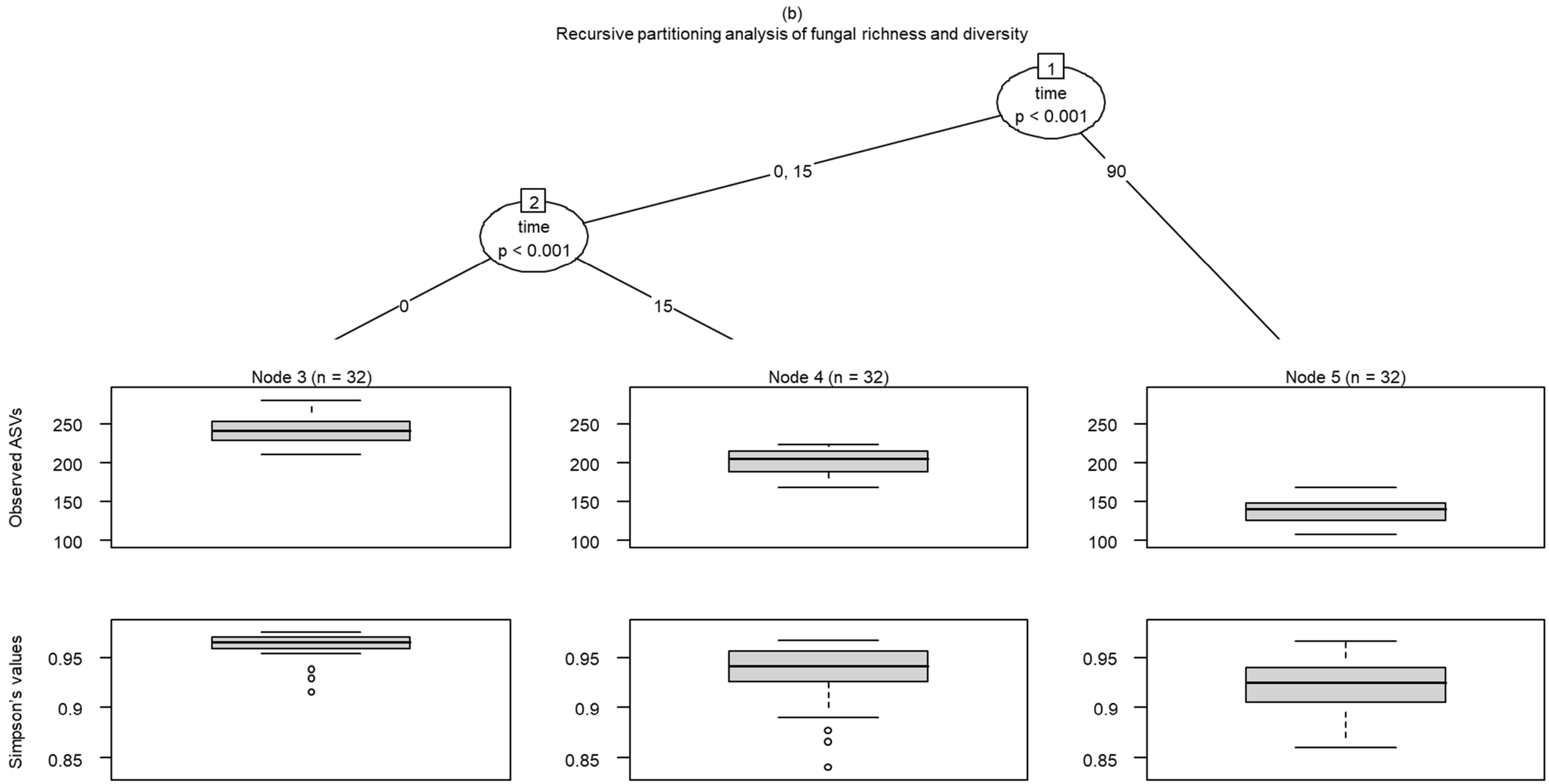

(b)
Recursive partitioning analysis of fungal richness and diversity
1
time
p < 0.001
0, 15
90
2
time
p < 0.001
0
15
Node 3 (n = 32)
Node 4 (n = 32)
Node 5 (n = 32)
Observed ASVs
100
150
200
250
Simpson's values
0.85
0.9
0.95

Figure S1: Recursive partitioning analysis of bacterial (a) and fungal (b) richness and diversity
